# Obese patients’ dissatisfaction with weight, body image and clinicians’ interaction at a district hospital; Gauteng

**DOI:** 10.4102/phcfm.v15i1.3872

**Published:** 2023-07-07

**Authors:** Buhendwa Kanozire, Deidré Pretorius

**Affiliations:** 1 Department of Family Medicine and Primary care, School of Clinical Medicine, University of the Witwatersrand, Johannesburg, South Africa; 2 Division of Family Medicine, School of Clinical Medicine, University of the Witwatersrand, Johannesburg, South Africa

**Keywords:** obese patient, weight, body image, dissatisfaction, clinicians, obesity management

## Abstract

**Background:**

Obesity in South Africa has created a public health crisis that warrants a multilevel intervention. However, patients’ perceptions and clinicians’ challenges hinder the management of obesity in primary care.

**Aim:**

The study aimed to assess obese patients’ dissatisfaction with weight and body image and their perspectives on interaction with clinicians regarding obesity management in a primary care setting.

**Setting:**

Outpatient department of Dr Yusuf Dadoo District Hospital.

**Methods:**

Cross-sectional study of 213 adult obese patients. A semi-structured questionnaire, a body image assessment tool and patients’ medical records were used for data collection.

**Results:**

The study found that, contrary to popular belief, obese patients were dissatisfied with their weight (78.9%) and body image (95.3%). Many felt comfortable while discussing weight reduction with clinicians, although 37.1% reported never engaging with a doctor and 62.9% never interacted with a nurse on the subject. Only 6% reported receiving adequate information on weight reduction measures and 19.7% were followed-up. Clinicians’ advice was mainly associated with patients’ high body mass index and waist circumference. Doctors were less likely to recommend weight reduction to employed obese women, while nurses were more likely to engage Zulu-speaking patients. Patients were more likely to be followed up if they were young and excessively obese.

**Conclusion:**

The study found that most obese patients were dissatisfied with their weight and body image and perceived their interaction with clinicians regarding obesity management as inadequate.

**Contribution:**

The study provides an angle of view of challenges in obesity management from patients’ perspectives.

## Introduction

### Background

Obesity is an important contributing risk factor to many non-communicable diseases (NCDs), an induction factor for multimorbidity and a challenge for patients and clinicians in primary care practice.^1,2^ In 2019, overweight and obesity cost the South African economy about 4.16% of the gross domestic product (GDP) or R219.15 billion.^3^ Prevention and control of overweight and obesity are essential in reducing the burden of NCDs on strained healthcare systems, especially in countries with limited resources.^2,4^

The SANHANES-1 report had previously brought to light the crisis level of NCDs and obesity in South Africa,^5^ and according to the 2016 South African statistics on health survey, about two-thirds of women and one-third of men in the country were overweight or obese.^6^ At this level of prevalence, obesity in South Africa has undoubtedly become an epidemic with no downtrend in sight if serious measures are not taken to tackle this significant public health crisis.^2,7,8^ As is the case in all epidemics, experts have advocated a multilevel approach to preventing and controlling obesity. This approach involves key players, such as government policymakers, clinicians and patients and their communities, who need to play their roles decisively.^9,10^

Effective government policy implementation is necessary, but as it has been the case in many other countries, the South African government has recognised the negative impact of obesity on public health and its strain on the health system. Nonetheless, it is still struggling to find the best suitable strategy for preventing and controlling overweight and obesity.^9,11,12^

The role of primary care providers in preventing and controlling overweight and obesity cannot be over-emphasised as they are at the forefront and constitute a key determinant factor in the fight against obesity.^13,14,15,16^ However, studies have shown that many clinicians experience difficulty integrating weight management into their routine practice.^17,18,19^ Some cannot identify obesity, while others poorly document obesity as a patient’s problem that they need to address.^20,21^ Some clinicians are biased and demonstrate a negative attitude regarding obese patients’ ability or willingness to self-discipline or effectively engage with prescribed measures.^22^ In contrast, others express ambiguity concerning the effectiveness of existing obesity management models.^23^

These clinicians’ deficiencies significantly hinder their ability to fulfil their role and limit their contribution to the fight against overweight and obesity. However, obese patients and their communities contribute to the challenges faced by clinicians in managing obesity at the primary care level. The patient’s role and that of their community are crucial for the optimal implementation of measures aimed at preventing and controlling obesity. A weight loss management plan can only be successful if there is buy-in from the patients and the communities that support them.^20^ This is even more relevant to the South African context, as studies have demonstrated the impact culture has on individuals’ and communities’ perceptions of weight in the country.^24,25^ It has been shown that many obese patients see themselves as normal, content with excessive weight and show no interest in weight reduction measures.^26,27^ This phenomenon is prevalent in patients whose cultural environment has normalised obesity. One highlight of such an environment is the emergence of the body positivity movement worldwide, which critics regard as a counterproductive factor in the fight against obesity.^28^ Other obese patients demonstrate poor insight into the health risk associated with obesity and show little appetite to embark on weight reduction measures despite being advised to do so.^26,28^ However, recent studies have reported a gradual shift in obese patients’ perception of health risks associated with obesity as more and more patients recognise the need for weight reduction measures.^26,29,30^ Unfortunately, the lack of motivation, the limited knowledge of weight-loss strategies and the limited access to resources and professional assistance continue to hinder their efforts.^26,29,30^

Obese patients’ dissatisfaction with their weight and body image remains one of the determinant factors in their decision to participate in weight control measures.^30,31^ Although a small proportion of obese patients will attempt to lose weight, studies have shown that few will manage to maintain an ideal weight if professional support is not provided.^32,33^ The challenges faced by obese patients in controlling their weight have been partly attributed to a lack of constructive interaction with clinicians.^14,15,19,34^

In recent years, within South Africa, few studies have explored obese patients’ views on their interaction with clinicians in primary care settings, and no study has so far been carried out in the West Rand. This study aimed to assess obese patients’ dissatisfaction with weight and body image and their perspectives on interactions with clinicians regarding obesity management in a primary care setting. Its objectives were to firstly determine the socio-demographic characteristics of obese patients attending the district hospital’s outpatient department, secondly to determine obese patients’ dissatisfaction with their weight and body image and how comfortable they were discussing weight management with clinicians, thirdly to evaluate obese patients’ views on their interaction with clinicians regarding weight reduction advice and factors that influence clinicians’ provision of advice for weight reduction and follow-up.

## Research methods and design

### Study design

This was a descriptive, observational cross-sectional study.

### Study setting

This study was conducted in the outpatient department at Dr Yusuf Dadoo District Hospital, located in Mogale City, west of Gauteng. Several primary healthcare centres feed the hospital, and its catchment area comprises a low and middle-income population of about 649 751 with mixed ethnicity from surrounding suburbs and townships. The languages most spoken in the area are Tswana, Afrikaans and Zulu.

### Study population

The study population included obese patients aged 18 years and above attending Dr Yusuf Dadoo District Hospital Outpatient Department. The World Health Organization’s (WHO) definition criteria of obesity (body mass index [BMI] of 30 kg/m^2^ and more or a waist circumference [WC] of 102 cm or more for men and 88 cm^2^ or more for women)^35^ was used for sample selection. Patients were excluded if they were very ill, pregnant or had an ongoing unstable psychiatric condition.

### Sampling strategy

In 2016, the WHO reported that 28.3% of the adult population in South Africa was obese.^36^ As the study was conducted in a predominantly chronic outpatient department, the sample was adjusted to a finite population of 680, corresponding to the average number of chronic patients seen monthly in the district hospital’s outpatient department. The calculated sample size was 213 based on the above-estimated population proportion with a confidence level of 95% and a margin error of 0.05.

### Selection of participants

Convenient sampling of consecutive patients was used for the selection of participants. The principal investigator collaborated with the nursing staff at the outpatient department. There was no systematic randomisation and patients were invited to the study as they presented for collection of vital signs and fulfilled the inclusion criteria.

### Data collection tool

The data collection tool had four sections. The first comprised anonymised respondents’ demographic details. The second contained a semi-structured questionnaire related to the patient’s dissatisfaction with weight, how comfortable they were, discussing weight reduction measures with a clinician, and their perspectives of interaction with clinicians regarding weight reduction advice and subsequent follow-ups. The questions used to construct our questionnaire were originally part of a focus group session guide used in a study by Thomas and colleagues.^37^ Even though the study was described as qualitative, other parameters in the study point to a mixed method and its focus group questionnaire was well-tailored to answer our primary research questions after adaptation. As part of the adaptation, the authors changed the body image assessment tool used in the study to a more culturally relevant and gender inclusive one. The questionnaire was piloted to test its feasibility and relevance in our study setting, leading to some amendments and additions. Also, as a result of the pilot study, the questionnaire was translated by the study supervisor into Afrikaans to accommodate the large proportion of Afrikaans-speaking participants who could not be assisted by the research assistant, who was more fluent in Tswana and Zulu. The piloted number was not added to the final sample because of the substantial amendments and additions. The third section of the data collection tool contained a body image assessment tool, a validated tool developed as a culturally relevant instrument for measuring body image dissatisfaction in males and females.^38^ It is a figural scale that comprises two sets of nine silhouettes numbered from one to nine. One represents a perceived thin body image and nine is an extremely fat body. Participants were invited to first circle the number of the silhouette they perceive to be their current body image (first set) and later the number corresponding to the silhouette of what they perceive to be their ideal body image (second set). The difference between these rankings indicates an existing body image dissatisfaction, usually related to a slim ideal.^38^ The last section comprised a recording sheet for biometric data, namely anthropometric measurements (weight, height and WC), calculated BMI and any ongoing NCD the patient suffered from during the study. The WHO recommended BMI cut-offs and protocol for WC measurement (Midpoint between the lower edge of the rib cage and the crest of the ilium) were used to assess the patient’s anthropometry and define obesity.^39^

### Data analysis

Collected data were computed in Microsoft Excel. Each categorical variable was coded based on a pre-defined code sheet arranged by the principal investigator to minimise errors before uploading it into Stata Statistical Software version 16.1, 2019 release for analysis. Continuous variables were reported as median and ranges because they were not normally distributed and categorical variables as frequencies and percentages. The International Atherosclerosis Society (IAS) and International Chair on Cardiometabolic Risk (ICCR)’s consensus statement on WC were used to categorise WC by cardiovascular disease (CVD) risk.^40^ To measure body image dissatisfaction, the authors calculated the absolute difference between the numbers corresponding to the current perceived body image and the ideal body image on the body assessment tool. Any result above one indicated dissatisfaction and any below suggested no dissatisfaction. Obese patients’ perspectives on their interaction with clinicians were assessed using reports of advice on weight reduction and follow-up after the initial discussion. Chi-squared tests were performed to determine any association between patients’ characteristics and their interaction with clinicians. A two-step logistic regression was performed and its results are reported as odds ratios (OR), confidence intervals (95% CI) and *p-*values (*p*). Univariate logistic regression models were conducted to assess whether patients’ characteristics influenced clinicians’ advice on weight reduction and subsequent follow-up. Multivariate logistic regression models were performed to control confounders by adjusting statistically and clinically significant factors. A *p*-value < 0.05 was deemed statistically significant for variable entered into the model.

### Ethical considerations

The study proposal was submitted to the Wits Human Research Ethics Committee for ethics clearance and was approved under the protocol reference number M200118. Permission to conduct the study was also obtained from the National Human research Council-Gauteng (NHRD_ GP_202003_015) – M200118; and Dr Yusuf Dadoo’s hospital management. All study participants provided written consent and were informed of their participation’s voluntary nature and their right to withdraw at any time with no impact on the service they ought to receive. The data collection sheet and questionnaire were anonymised.

## Results

Data collection was conducted over a period of 3 months. A total of 230 patients were eligible for participation in the study. Five severely ill and two pregnant patients were excluded. Altogether 223 patients were invited, but 10 declined, leaving 213 participants. No reason for the refusal was asked, as it was the patient’s right to refuse.

### Socio-demographic characteristics of patients

Obese patients’ baseline characteristics (Table 1) show that the median age was 53 years, ranging from 23 to 75 years. Most obese patients attending the hospital’s outpatient department during the study were aged from 46 to 55 years (67; 31.5%) and 56 to 65 years (63; 29.6%). There were more women (153; 71.8%) than men (60; 28.2%). Most were Tswana speaking (69; 32.4%), followed by Afrikaans (59; 27.7%). A total of 87 patients were married (40.8%) and 147 (69%) had completed secondary school. A total of 135 patients were employed (58.7%). There were 168 (78.9%) severely obese patients with BMIs of 35 kg/m^2^ or above and 85 (39.9%) were extremely obese (BMI ≥ 40 kg/m^2^). Cardiovascular disease risk stratification related to WCs showed that 169 (79.34%) patients had extremely high CVD risk. A total of 87 patients (40.8%) had a metabolic disease, and 73 (34.27%) were multimorbid.

**TABLE 1:** Sociodemographic characteristics of patients (*n* = 213).

Baseline characteristics	Frequency	Percentage
**Age (years)†**		
18–25	1	0.5
26–35	19	8.9
36–45	36	16.9
46–55	67	31.5
56–65	63	29.6
>65	27	12.7
**Gender**		
Women	153	71.8
Men	60	28.2
**Language**		
Tswana	69	32.4
Afrikaans	59	27.7
Zulu	25	11.7
Sotho	14	6.6
Xhosa	18	8.5
Sepedi	6	2.8
Ndebele	5	2.4
English	10	4.7
Venda	7	3.3
**Marital status**		
Single	33	15.5
Married	87	40.9
Divorced	31	14.6
Living together	29	13.6
In relationship	10	4.7
Widowed	23	10.8
**Education**		
No formal education	8	3.8
Primary school	28	13.2
Secondary school	147	69
Tertiary education	30	14.1
**Employment status**		
Employed	125	58.7
Unemployed	54	25.5
Retired	34	16
**BMI (kg/m^2^)‡**		
Class 1 (30–30.9)	45	21.1
Class 2 (35–39.9)	83	39
Class 3 (> 40)	85	40
**WC (cm)§**		
Very high CVD risk¶	44	20.7
Extremely high CVD risk††	169	79.3
**Comorbidities**		
Metabolic disease	87	40.9
Multimorbidity*	73	34.3
Cardiovascular	36	16.9
Respiratory diseases	8	3.8
Musculoskeletal disease	3	1.4
Cancer	2	0.9
Other	4	1.9

BMI, body mass index; WC, waist circumference; CVD, cardiovascular disease.

* Multimorbidity refers to the patient suffering from two or more non-communicable diseases other than a metabolic syndrome.

† , Median: 35 (23–75);

‡ , Median: 38.5 (31.5–63.7);

§ , Median: 126 (107–173);

¶ , (Female ≥ 105; Male ≥ 110);

†† , (Female ≥ 115; Male ≥ 125).

Most obese patients reported a great level of dissatisfaction with their weight and body image (Table 2). Of these, 168 (78.9%) expressed unhappiness with their current weight, 199 (93.4%) had thought of losing weight, 156 (73.2%) reported previous weight loss attempts, and 203 (95.3%) expressed dissatisfaction with their current body image.

**TABLE 2:** Obese patients’ level of dissatisfaction with weight and body image (*n* = 213).

Variables	Responses	Frequency	Percentage
Current feelings about weight	Happy	45	21.1
	Not happy	168	78.9
Ever thought of losing weight	No	14	6.6
	Yes	199	93.4
Ever attempted to lose weight	No	57	26.8
	Yes	156	73.2
Dissatisfied with body image	No	10	4.7
	Yes	203	95.3

Table 3 documents obese patients’ reported comfort while discussing weight management and interaction with clinicians. Altogether, 197 (92.5%) patients were comfortable discussing weight management with a doctor, and 210 (98.6%) were happy discussing it with a nurse. A total of 79 (37.1%) patients reported never being engaged by a doctor and 134 (62.9%) never engaged with a nurse. A total of 111 (52.1%) obese patients who were either engaged by a doctor or a nurse reported not receiving information on how to lose weight and 89 (41.8%) thought they were not given enough information. A total of 171 (80.3%) patients said no clinician followed up with them after an initial weight loss discussion.

**TABLE 3:** Obese patients’ reported comfort in discussing weight management and interaction with clinicians (*n* = 213).

Variables	Responses	Frequency	Percentage
Comfortable discussing weight management with a doctor	No	16	7.5
	Yes	197	92.5
Comfortable discussing weight management with a nurse	No	3	1.4
	Yes	210	98.6
Have been told by a doctor to lose weight	Never	79	37.1
	Yes	134	62.9
Have been told by a nurse to lose weight	Never	134	62.9
	Yes	79	37.1
Information received on how to lose weight	None	111	52.1
	Not enough	89	41.8
	Enough	13	6.1
Followed-up after weight loss discussion	Never	171	80.3
	Yes	42	19.7

### Association between obese patients’ characteristics and interactions with clinicians

Chi-squared test results showed that doctors’ provision of advice on weight loss was significantly associated with patients’ BMI (*p* < 0.001) and WC (*p* < 0.001). In contrast, nurses’ advice was associated with patients’ language (*p* = 0.007), BMI (*p* = 0.004) and WC size (*p* = 0.01). A significant association was also found between the amount of information received for weight reduction and patients’ age (*p* = 0.013), BMI (*p* < 0.001) and WC (*p* < 0.001). There was no association between obese patients’ medical conditions and the information received (*p* = 0.89). Follow-up of patients after the initial provision of advice on weight reduction was significantly related to the patient’s age (*p* = 0.006), BMI (*p* = 0.001) and WC size (*p* = 0.004).

### Factors influencing clinicians’ provision of advice on weight reduction

Results of logistic regressions (Table 4) suggest that patients were more likely to be advised by doctors to lose weight if they were morbidly obese (Class 3 BMI) (OR = 4.5; 95% CI: 2.03–10.02; *p* = 0.001) and had a severely large WC (OR = 3.6; 95% CI: 1.79–7.12; *p* < 0.001). After adjusting for age, sex and medical conditions, results demonstrate that high BMI and severely large WC still positively influenced doctors advising weight loss (OR = 3.6; 95% CI: 1.34–9.88; *p* = 0.011) and (OR = 4.714; 95% CI: 1.64–13.58; *p* = 0.004). While controlling for occupation, BMI and WC, women became less likely to be advised by a doctor to lose weight (OR = 0.42; 95% CI: 0.18–0.97; *p* = 0.042).

**TABLE 4:** Univariate logistic regression modelling advice by doctor and nurse to lose weight as well as clinicians’ follow-up of patients.

Patients’ characteristics	Advised by doctor to lose weight			Advised by nurse to lose weight			Clinicians’ follow-up		
	OR	95% CI	*p*-value	OR	95% CI	*p*-value	OR	95% CI	*p*-value
**Age**									
18–25	Ref	-	-	Ref	-	-	Ref	-	-
26–35	0.619	0.189–2.020	0.427	3.175	0.913–11.033	0.069	18.909	2.105–169.826	0.009
36–45	1.080	0.390–2.992	0.882	2.041	0.689–6.047	0.198	3.25	0.342–30.884	0.305
46–55	1.506	0.597–3.798	0.386	1.494	0.551–4.050	0.431	7.5	0.939–59.929	0.057
56–65	1.196	0.475–3.010	0.704	1.643	0.603–4.474	0.331	6.76	0.837–54.568	0.073
**Gender**									
Female	Ref	-	-	Ref	-	-	Ref	-	-
Male	1.026	0.552–1.904	0.936	1.188	0.643–2.193	0.582	1.132	0.527–2.429	0.750
**BMI**									
Class 1 (30–30.9)	Ref	-	-	Ref	-	-	Ref	-	-
Class 2 (35–39.9)	1.124	0.544–2.322	0.753	0.944	0.422–2.109	0.887	0.791	0.262–2.383	0.676
Class 3 (>40)	4.508	2.029–10.018	<0.001	2.520	1.164–5.455	0.019	3.026	1.143–8.008	0.026
**WC**									
Very high CVD risk	Ref	-	-	Ref	-	-	Ref	-	-
Extremely high CVD risk	3.573	1.793–7.118	0.000	2.749	1.243–6.082	0.013	6.512	1.509–28.1	0.012
**Language**									
Tswana	Ref.	-	-	Ref.	-	-	Ref	-	.
Afrikaans	1.163	0.566–2.389	0.68	1.457	0.694–3.056	0.32	0.9	0.374–2.169	0.815
Zulu	2.036	0.721–5.744	0.179	6.3	2.28–17.405	0.00	1.241	0.417–3.687	0.698
Sotho	0.857	0.268–2.744	0.795	0.668	0.168–2.652	0.566	0.302	0.036–2.51	0.268
Xhosa	0.804	0.282–2.291	0.683	0.942	0.297–2.991	0.92	1.511	0.461–4.95	0.495
Sepedi	1.286	0.220–7.51	0.78	4.9	0.83–28.919	0.079	3.929	0.714–21.601	0.116
Ndebele	0.964	0.151–6.153	0.969	1.633	0.253–10.526	0.606	1	-	.
English	0.964	0.249–3.736	0.958	1.633	0.416–6.414	0.482	0.982	0.187–5.149	0.983
Venda	0.857	0.178–4.133	0.848	0.408	0.046–3.612	0.421	1	-	.
**Marital status**									
Single	Ref	-	-	Ref	-	-	Ref	-	-
Married	1.370	0.597–3.143	0.458	0.509	0.226–1.145	0.103	0.696	0.276–1.755	0.442
Divorced	0.693	0.257–1.869	0.469	0.290	0.101–0.834	0.022	0.513	0.151–1.747	0.286
Living together	1.063	0.382–2.964	0.906	0.439	0.157–1.226	0.116	0.556	0.162–1.902	0.349
In relationship	1.517	0.331–6.949	0.592	0.556	0.132–2.342	0.423	0.296	0.033–2.683	0.279
Widowed	1.011	0.340–3.008	0.984	0.294	0.093–0.934	0.038	0.561	0.150–2.107	0.392
**Education level**									
No education	Ref	-	-	Ref	-	-	Ref	-	-
Primary school	2.111	0.428–10.423	0.359	1.667	0.282–9.856	0.573	0.538	0.042–6.837	0.633
Secondary school	1.579	0.380–6.565	0.530	1.691	0.330–8.679	0.529	1.795	0.213–15.154	0.591
College and/or Tertiary	2.333	0.475–11.451	0.297	2.625	0.454–15.162	0.281	3	0.321–28.069	0.336
**Employment status**									
Employed	Ref	-	-	Ref	-	-	Ref	-	
Unemployed	2.035	1.006–4.119	0.048	0.814	0.419–1.581	0.544	0.633	0.278–1.445	0.278
Retired	1.15 1	0.529–2.505	0.724	0.62 5	0.275–1.419	0.261	0.306	0.087–1.074	0.065
**Comorbidities**									
Cancer	Ref	-	-	Ref	-	-	Ref	-	
Cardiovascular	1.118	0.65–19.283	0.939	0.636	0.080–5.050	0.669	0.724	0.065–8.054	0.793
Metabolic disease	2.346	0.141–38.952	0.552	0.851	0.115–6.319	0.875	0.676	0.066–6.929	0.742
Musculoskeletal disease	0.5	0.013–19.562	0.711	-	-	-	-	-	
Multimorbidity	1.92	0.115–32.008	0.650	0.431	0.057–3.261	0.415	0.911	0.089–9.335	0.937
Respiratory diseases	0.333	0.014–8.182	0.501	0.143	0.008–2.517	0.184	0.429	0.020–9.364	0.590

CI, confidence interval; OR, odds ratio; BMI, body mass index; WC, waist circumference; CVD, cardiovascular disease.

Patients who spoke Zulu were 6.3 times more likely to be advised by nurses to lose weight (OR = 6.3; 95% CI: 2.28–17.41; *p* < 0.001) than their counterparts. Those who had morbid obesity were 2.5 times more likely (OR = 2.5; 95% CI: 1.16–5.46; *p* = 0.019) and those with severely large waist sizes were 2.8 times more likely to be advised by nurses to lose weight (OR = 2.8; 95% CI: 1.243–6.082; *p* = 0.013). The results also show that divorced and widowed patients were less likely to be advised by nurses to lose weight (OR= 0.29; 95% CI: 0.1–0.83; *p* = 0.022) and (OR = 0.29; 95% CI: 0.09–0.93; *p* = 0.038). These factors remain unaffected after controlling for age, sex, education, employment status and medical condition.

### Factors influencing clinicians’ follow-up of obese patients after prior advice for weight reduction

Results of univariate analysis showed that young age, BMI and WC were statistically significant in influencing clinicians’ follow-up of obese patients (Table 4). When controlling these significant variables with the inclusion of gender for clinical significance, age and WC are the only factors affecting follow-up (Table 5). The univariate model analysis (Table 4) showed that young patients aged 26–35 were 18.9 times more likely to be followed up after initial advice to lose weight than their counterparts (OR = 18.9; 95% CI: 2.1–169.8; *p* = 0.009), patients with BMI > 40 were three times more likely to be followed up after initial advice to lose weight (OR = 3.026; 95% CI: 1.143–8.008; *p* = 0.026), and those with extremely large WC were 6.5 more likely to be followed up (OR = 6.5; 95% CI: 1.509–28.1, *p* = 0.012). In the multivariate analysis (Table 5), Body mass index ceased to be a significant factor for follow-up (OR = 1.976; 95% CI: 0.66–5.87; *p* = 0.22), while WC remained a significant factor, with those with very large WC 3.6 times more likely to be followed up (OR = 5.21; 95% CI: 1.012–26.919; *p* = 0.048).

**TABLE 5:** Multivariable logistic regression modelling of clinicians’ follow-up of patients.

Patients’ characteristics	Clinicians’ follow-up		
	OR	95% CI	*p*
**Age**			
18–25	Ref.	-	-
26–35	18.891	1.975–180.69	0.011
36–45	2.266	0.226–22.664	0.486
46–55	5.818	0.702–48.255	0.103
56–65	5.464	0.651–45.864	0.118
> 65	Ref.	-	-
**Sex**			
Male	Ref.	-	-
Female	0.461	0.184–1.157	0.099
**BMI**			
Class 1 (30–30.9)	Ref.	-	-
Class 2 (35–39.9)	0.679	0.208–2.213	0.521
Class 3 (>40)	1.976	0.665–5.868	0.22
**WC**			
Very high CVD risk	Ref.	-	-
Extremely high CVD risk	5.219	1.012–26.919	0.048

CI, confidence interval; OR, odds ratio; BMI, body mass index; WC, waist circumference; CVD, cardiovascular disease.

## Discussion

This study aimed to assess obese patients’ dissatisfaction with weight and body image and their perspectives on interaction with clinicians in a primary care setting. The findings demonstrate that most obese patients attending Dr Yusuf Dadoo District Hospital’s outpatient department were middle-aged adult women. Most of the patients spoke Tswana and Afrikaans, had completed secondary school and were employed. A large proportion of patients were married, severely obese, and demonstrated a very high level of CVD risk. The majority either suffered from metabolic disease or were multimorbid. These findings are broadly consistent with the 2016 Statistics South Africa report on health surveys concerning obesity.^6^ Although that survey did not account for the employment and marital status of obese individuals, it showed that obesity was associated with a high level of wealth. These findings are also consistent with trends previously reported in South Africa and other parts of the world.^6,7,41^

Popular beliefs and previous studies showed that larger body size is socially or culturally accepted and has been normalised in many parts of South Africa and the world.^29,30^ In sharp contrast, this study’s findings show that most obese patients reported unhappiness with their current weight and their perceived body image. Many stated they had thought of losing weight, and a significant proportion reported having attempted weight reduction. These findings, for example, partly diverge from the SANHANES-I report on weight management and body image,^5^ which showed that most South Africans were happy with their body size. However, only 42% of the study participants correctly linked their desired body size with their perceived one,^5^ thus demonstrating a greater level of body image dissatisfaction. The discrepancy between this study’s results and those previously reported in the literature could be explained by increased public awareness of the risk associated with obesity and the desire to stay fit and healthy.^29,30,31^ Also, the emerging shift in attitude to excessive weight as more people are exposed to western media that portrays slimness as ideal and fashionable^42^ may explain this possible shift. A good example is the emerging popularity of ‘fitspiration’ or ‘fitness inspiration’, promoting healthy living through exercise and a healthy diet.^43^ However, a counterargument to this is the increase in the number of social media exposés that support the body positivity movement, which promotes the acceptance of each body type and size,^28^ although critics consider this as a way of promoting obesity and unhealthy behaviour. This ambiguity in the social media environment has been recently highlighted.^28,43,44^

The study results show that most patients were comfortable discussing weight reduction measures with clinicians, often with nurses, than with doctors. However, they mostly received advice from doctors and less from nurses, demonstrating an underutilisation of the full potential of nurse-led intervention in the fight against obesity at the primary care level, which has shown to be effective in other parts of the world.^45^

Results also showed that even though clinicians recommended weight loss to all the patients in the study in one way or another, only 6% of the patients thought the information received was adequate. Most patients either received no information or found it insufficient to help them succeed with weight loss. In addition, the amount of information received was significantly associated with the patient’s age, BMI and WC. These findings highlight discrepancies between obese patients’ expectations of the clinicians’ role and the level of assistance they receive in clinical practice.^14,23,46^ They further demonstrated the need for clinicians to adjust their attitude to obese patients’ willingness to engage in weight reduction measures and to consider improving the quality of care provided to the obese population.^22,23,47^

The study results suggested that clinicians’ provision of advice for weight reduction was significantly associated with morbid BMI and severely large WC. These findings corroborate previous reports, showing that primary care clinicians tended to manage obesity proactively while clinicians perceived patients to be in a morbid state or had accumulated extremely severe CVD risk.^17^ It has also been found that clinicians often underestimate their role in preventing and controlling overweight and obesity.^14,48,49^ While adjusting BMI and WC with employment status, employed obese women were less likely to be advised by doctors to lose weight. This contrasts sharply with previous findings, suggesting that patients with better socio-economic status were more likely to engage healthcare providers about weight management.^50^ It has also been shown that compared with men, employed obese women are more likely to be absent from work because of weight-related problems.^51^ The researchers speculate that the study’s finding of discriminatory attitude on the part of the doctors towards employed, obese women could be explained by the high ratio of male to female doctors who have to engage a majority of female patients on a sensitive subject such as obesity.^52^ However, further studies are needed in this regard. Nurses’ advice was also strongly related to the patient’s language and marital status. They were 6.3 times more likely to advise patients who spoke Zulu and less likely if patients were divorced or widowed. The researchers speculate that this finding can be attributed to previously reported bias clinicians demonstrate in managing obesity.^53,54^ However, they could also highlight the need for tailored, culturally sensitive interventions in managing obesity. Several authors have previously advanced this argument, although others found this had little effect on obesity prevention and control.^55,56^

This study found that only one out of five patients who were advised to lose weight reported having been followed up, and this follow-up was more likely to occur if patients were young and morbidly obese. This study further demonstrated that, while clinicians could initiate discussion on weight reduction, the benefit to obese patients was minimal because of poor information and a lack of follow-up needed to enhance adherence to prescribed weight reduction measures. Previous studies have attributed clinicians’ inability to provide adequate information and follow-up of obese patients to their biased attitude, limited knowledge, poor understanding of their role and deficiencies in the healthcare systems.^14,18,19,33^

The literature suggests that clinicians’ and obese patients’ challenges in addressing obesity in primary care can be because of multiple factors, such as time constraints, clinicians’ and patients’ attitudes towards obesity, clinicians’ level of knowledge and experience and the availability of resources.^17,18,19,20,21,22,23,24^ The findings in this study have highlighted previously reported trends in managing obesity at the primary care level. However, it has also brought some new results that contradict previous beliefs and others that warrant further studies.

### Strengths and limitations

This is the first study in West Rand exploring the relationship between obese patients’ dissatisfaction with their weight and body image and their perspective of interaction with clinicians regarding obesity management at the primary care level. It may be the first of its kind in South Africa. The study focused on the frequency of reported patients’ weight and body image dissatisfaction and did not account for the reasons for dissatisfaction. There was possibly a selection bias because of the convenience sampling strategy. To minimise its effect on reliability, the nursing team invited every consecutive patient who responded to the inclusion criteria. While information or social desirability bias could have affected the reliance on patients’ reported information, the researcher and research assistant ensured that patients’ anonymity and confidentiality were always kept, minimising self-reporting bias during data collection. Although the study demonstrates a high prevalence of weight and body image dissatisfaction in the obese population attending the district hospital, these findings need to be interpreted in the context of a mixed cultural divide because ethnicity has been found to affect obesity perception.^57,58,59^ Also, while the study’s findings might suggest a societal shift in obesity perception, this needs to be tested in more settings.

Obese patients’ perceived inadequacies in their interaction with clinicians can be improved by training clinicians on obesity awareness, communication skills and strategic approach to weight loss intervention at the primary care level and by ensuring that a more patient-centred approach is applied during clinical encounters. The district hospital’s clinical team should implement a contextualised clinical guideline based on the national strategic approach for controlling and preventing obesity to assist clinicians in their daily practice. The diverging high rate of weight and body image dissatisfaction among obese patients in the study compared with previous reports might signal a social shift in obesity perception that warrants large-scale investigations such as a second SANHANES study. Also, further studies are required to explore why doctors are less likely to recommend weight loss to employed, obese women.

## Conclusion

This study’s findings have shown that, contrary to popular belief, most obese patients are dissatisfied with their weight and body image. However, they perceived their interaction with clinicians at Dr Yusuf Dadoo District Hospital inadequate for the level of help they needed to achieve satisfactory weight loss. The study results also demonstrate that obesity management in primary care demands further clinician education.
